# Research on RNA modification in disease diagnosis and prognostic biomarkers: current status and challenges

**DOI:** 10.1093/bib/bbaf361

**Published:** 2025-07-23

**Authors:** Hua Shi, Zhouying Li, Quan Zou, Hui Yang

**Affiliations:** School of Opto-electronic and Communication Engineering, Xiamen University of Technology, Ligong Road, Jimei District, Xiamen, Fujian 361024, China; School of Opto-electronic and Communication Engineering, Xiamen University of Technology, Ligong Road, Jimei District, Xiamen, Fujian 361024, China; Yangtze Delta Region Institute (Quzhou), University of Electronic Science and Technology of China, Chengdian Road, Kecheng District, Quzhou, Zhejiang 324000, China; Institute of Fundamental and Frontier Sciences, University of Electronic Science and Technology of China, Section 2, North Jianshe Road, Chenghua District, Chengdu, Sichuan 610054, China; Yangtze Delta Region Institute (Quzhou), University of Electronic Science and Technology of China, Chengdian Road, Kecheng District, Quzhou, Zhejiang 324000, China

**Keywords:** RNA modifications, biomarkers, disease diagnosis, prognostic models, machine learning

## Abstract

RNA modification, as a crucial post-transcriptional regulatory mechanism, plays a pivotal role in normal physiological processes and is closely associated with the onset and progression of various human diseases. Recent studies have highlighted significant alterations in the level of RNA modifications, including m6A, m6Am, m1A, m5C, m7G, ac4C, Ψ, and A-to-I editing, across multiple diseases. These findings suggest the potential of RNA modifications and their regulatory factors as biomarkers for early disease diagnosis and prognosis. This review provides an overview of statistical methods, machine learning techniques employed in identifying disease diagnostic and prognostic biomarkers, along with relevant evaluation metrics and bioinformatics tools. We further explore the types of common RNA modifications, the modifying proteins involved, and the underlying mechanisms of modification. The focus of this paper is on the application of machine learning algorithms in discovering RNA modification-related biomarkers, particularly for disease diagnosis and prognosis. By reviewing recent advancements in the identification of disease biomarkers, and analyzing the prospects and challenges of their clinical application, we aim to offer insights into the mining methods of RNA modifications and their associated factors as disease diagnostic or prognostic biomarkers, providing a valuable reference for future research and clinical practice.

## Introduction

RNA modification refers to the post-transcriptional chemical changes in RNA molecules, also termed epitranscriptomic modifications. These modifications are essential for the dynamic regulation of gene expression. The epitranscriptome dynamically regulates the chemical modifications of both coding and non-coding RNA (ncRNA) molecules, which regulate the function and stability of RNA and thereby affect various biological processes in cells [1]. Proteins involved in RNA modifications, collectively known as RNA-modifying proteins, can be classified into three functional categories: ‘writers,’ ‘erasers,’ and ‘readers.’ Writers catalyze the addition of modifications, erasers remove them, and readers selectively recognize and bind to modified RNA.

Abnormalities in RNA modifications are closely linked to the initiation and progression of various diseases, particularly in cancer, neurological disorders, and cardiovascular diseases. Consequently, they can serve as biomarkers that reflect changes in specific diseases or physiological states, and they can also be used to assess the degree of body response to disease treatment [2]. For instance, in gastric cancer, m6A levels are significantly elevated compared to normal tissues [3]. The regulation of RNA modifications is a dynamic biological process, with modification levels exhibiting significant changes across diverse physiological and pathological conditions. Therefore, RNA modifications and their associated factors, including specific modification levels, RNA modification regulators, and modification-regulated genes, exhibit significant potential as biomarkers, offering novel approaches for early disease diagnosis and prognostic evaluation.

Methods for identifying biomarkers for disease diagnosis and prognosis include statistical methods, machine learning, bioinformatics tools, and experimental verification. Among these, machine learning algorithms enable the extraction of patterns and regularities from complex high-dimensional data, thereby generating knowledge and insights that facilitate prediction and decision-making [4]. In recent years, machine learning algorithms have been increasingly applied to the discovery of disease biomarkers. In particular, supervised learning methods have demonstrated significant potential in identifying RNA modification-based disease biomarkers, thereby enhancing the accuracy of disease diagnosis and prognostic prediction. The integration of RNA modification research with machine learning provides novel perspectives for advancing precision medicine diagnosis. However, their clinical translation still requires validation through independent cohort studies and investigations into molecular mechanisms.

This review summarizes the methods for identifying disease diagnostic and prognostic biomarkers based on RNA modification-related factors, encompassing statistical methods, machine learning algorithms, as well as bioinformatics tools. In addition, we introduce eight common types of RNA modifications along with their associated modifying enzymes or proteins, summarizes their regulatory mechanisms in diseases, with a focus on the research status of machine learning techniques in uncovering disease diagnostic and prognostic biomarkers related to RNA modifications. The aim of this review is to provide researchers with an overview of the current research status and insights into potential future directions regarding RNA modifications and their related factors as disease diagnostic and prognostic biomarkers. A literature search was conducted through platforms such as PubMed, limiting the scope to English-language publications from January 2020 to January 2025, focusing on studies related to RNA modifications and human disease diagnostic or prognostic biomarkers.

## Statistical methods, machine learning, and analytic tools

Statistical methods are employed to organize, summarize, generalize, and infer overall characteristics from data. Traditional statistical techniques, such as the t-test, analysis of variance (ANOVA), Mann–Whitney U test, Pearson’s correlation coefficient, and Spearman’s rank correlation coefficient, are designed to explore relationships between variables [5]. The t-test compares differences between two sample groups, while ANOVA is used to assess differences across multiple groups. In disease biomarker discovery, these methods can preliminarily identify biomarkers by comparing gene expression differences across groups or performing correlation analysis. Additionally, Cox regression is a widely used survival analysis model for evaluating the impact of variables, such as specific genes or other biomarkers, on patient survival [6]. Although traditional statistical methods are relatively easy to interpret, they rely on assumptions, such as the type of error distribution, which limits their applicability in analyzing complex data [5]. To overcome these limitations, various machine learning algorithms have been developed and increasingly applied to biomarker discovery.

**Figure 1:**
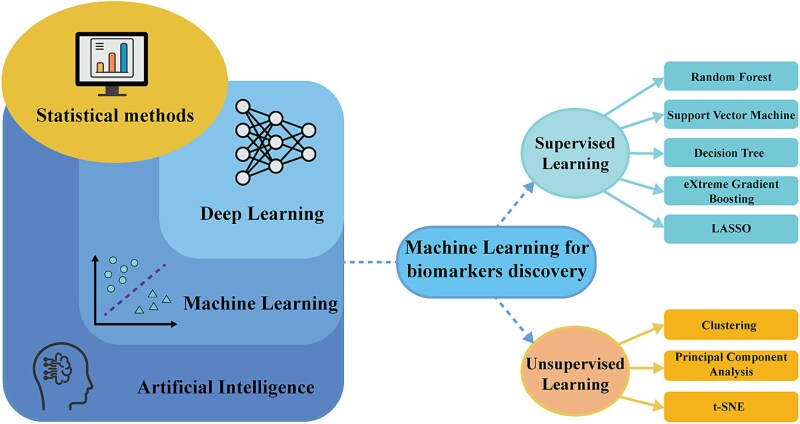
Relationships between AI, machine learning, deep learning, and statistical methods. AI algorithms include machine learning and deep learning algorithms. Machine learning algorithms commonly used to identify RNA modification-related disease biomarkers mainly include supervised learning and unsupervised learning.

Machine learning algorithms do not require a priori assumptions about the data, and they are capable of handling large-scale, diverse data distributions and high-dimensional datasets, and can identify complex patterns and learn nonlinear relationships within the data, offering greater flexibility [4, 7]. This flexibility provides machine learning methods with a significant advantage in biomarker discovery, particularly in the crucial step of feature selection. Machine learning algorithms can sift through numerous candidate features to identify potential disease biomarkers. Common machine learning techniques include supervised learning, unsupervised learning, semi-supervised learning, and reinforcement learning [8]. Among these, supervised and unsupervised learning are the most frequently employed in disease biomarker discovery. Supervised learning involves training models on labeled datasets and can be divided into classification and regression tasks [8]. Random Forest (RF) [9], Support Vector Machine (SVM) [10], Decision Tree (DT) [11], eXtreme Gradient Boosting (XGBoost) [12], and Least Absolute Shrinkage and Selection Operator (LASSO) [13] are commonly applied supervised learning algorithms in biomarker discovery (Fig. 1). Unsupervised learning seeks to uncover hidden structures and patterns in data without prior labels or information [14]. In biomarker discovery, unsupervised learning techniques are frequently employed to identify latent subtypes and extract salient features from complex datasets. For instance, clustering methods group samples with similar RNA modification patterns to distinguish groups of patients with different prognosis or treatment response [15]. Frequently used unsupervised learning algorithms include consensus clustering [16], principal component analysis (PCA) [17], and t-Distributed Stochastic Neighbor Embedding (t-SNE) [18] (Fig. 1). Material 2 in the Supplementary material provides an introduction to these algorithms.

Statistical and machine learning methods are both essential tools for feature selection, which serves as a critical step in disease biomarker discovery. By eliminating irrelevant variables, feature selection enhances the accuracy of biomarker identification. The primary strategies for feature selection include filter, wrapper, and embedded methods [19]. Filter methods utilize statistical tests or correlation analyses, such as t-tests and chi-square tests, to independently evaluate the relationship between each feature and the target variable. However, these methods may neglect interactions between features and considerations related to classifier choice. Wrapper methods iteratively evaluate feature combinations, such as linear SVM recursive feature elimination and genetic algorithms, based on the predictive performance of a given model. Embedded methods integrate feature selection directly into the model-building process by incorporating regularization penalties, such as LASSO, which automatically select relevant features during training. In addition, these embedded strategies can even be applied to less interpretable models, such as deep neural networks, to enhance feature selection. Thus, machine learning-based feature selection provides a robust foundation for identifying reliable and biologically meaningful RNA modification related biomarkers.

To evaluate the performance of biomarkers identified through feature selection, appropriate assessment metrics are required. Commonly used indicators for assessing evaluate diagnostic or prognostic biomarkers include area under the receiver operating characteristic (ROC) curve (AUC), sensitivity and specificity [8]. Among them, the ROC curve is a key tool for evaluating the diagnostic performance of biomarkers, showing the trade-off between the true positive rate (TPR) and the false positive rate (FPR) at different classification thresholds. In clinical applications, selecting the optimal threshold requires alignment with the biomarker’s intended purpose, as different scenarios prioritize different outcomes. For instance, in cancer screening, a lower threshold increases sensitivity, reducing false negative (FN) and ensuring more cases are identified. However, this comes at the cost of a higher false positive (FP) rate, potentially leading to misdiagnosis of healthy individuals. This trade-off is critical in early disease detection. Conversely, a higher threshold prioritizes specificity, minimizing FP but increasing the risk of missing true cases. To determine the optimal threshold, the Youden Index (J = TPR − FPR) is commonly used, selecting the threshold that maximizes the difference between correct and incorrect predictions. Additionally, cost-sensitive analysis incorporates domain-specific penalties for misclassification. For example, in malignant tumor screening, the high risk associated with delayed diagnosis makes FN more costly and therefore favors a lower threshold to minimize FN rates. Conversely, for diseases with low prevalence and high diagnostic cost of FP, a higher threshold is preferred to reduce FP [20]. Overall, the ROC curve is a valuable tool for assessing the impact of FP and FN on model performance, optimizing the threshold to balance sensitivity and specificity, and computing the AUC to evaluate the overall performance of the model. For prognostic biomarkers, in addition to prediction accuracy, survival analysis is necessary, incorporating the concordance index (C-index), Kaplan–Meier curves, hazard ratio (HR) and p-values. Material 3 in the Supplementary material provides descriptions and formulas for common performance evaluation metrics.

To ensure the reliability of selected biomarkers, a validation process is necessary to assess the model’s generalization ability. Validation methods can be categorized into internal and external verification. Internal validation typically involves techniques such as k-fold cross-validation or leave-one-out cross-validation, where the data is partitioned into training and testing sets to evaluate model performance. External validation includes evaluation of model performance using independent datasets and validation of biological functions of biomarkers through *in vivo* and in vitro experiments. For instance, quantitative real-time polymerase chain reaction (qRT-PCR) can be used to assess gene expression levels, while western blotting can verify changes in protein levels [21]. With external validation, the reliability of the biomarker can be tested and whether it is of value for further research can be determined.

Finally, bioinformatics tools are essential in disease biomarker discovery, enabling more precise and insightful analysis of identified biomarkers and the interpretation of their biological significance and mechanisms of action. Common tools include data statistical modeling tools, functional annotation tools, network analysis tools, and public database resources such as Gene Expression Omnibus (GEO) and The Cancer Genome Atlas (TCGA) for analysis. For instance, the Gene Ontology (GO) database divides the gene functions into three categories: cellular component, biological process and molecular function [22]. Kyoto Encyclopedia of Genes and Genomes (KEGG) enrichment analysis provides functional and signaling pathway for related molecules and metabolites within the context of disease [23]. The STRING database facilitates the exploration of functional proteins and protein–protein interactions linked to key genes [24]. Weighted gene co-expression network analysis (WGCNA) can identify modules of co-expressed genes, including those strongly correlated with disease phenotypes, and extract hub genes within these modules [25]. Numerous studies employ a variety of tools and online databases to analyze key genes [26–28].

## Common types of RNA modifications

Currently, over 170 distinct RNA modifications have been identified [1]. Common RNA modifications include N6-methyladenosine (m6A), N6,2’-O-dimethyladenosine (m6Am), N1-methyladenosine (m1A), 5-methylcytosine (m5C), N4-acetylcytosine (ac4C), N7-methylguanosine (m7G), Pseudouridine (Ψ), adenosine-to-inosine (A-to-I) editing, alternative polyadenylation (APA), and 2’-O-methylation (Nm). These modifications occur in a wide range of RNAs, including messenger RNA (mRNA), ribosomal RNA (rRNA), long non-coding RNA (lncRNA), transfer RNA (tRNA), microRNA (miRNA), circular RNA (circRNA), small nuclear RNA (snRNA), small nucleolar RNA (snoRNA), and enhancer RNA (eRNA) [1].

The regulation of RNA modifications is a complex and dynamic process mediated by specific proteins known as ‘writers,’ ‘erasers,’ and ‘readers,’ which catalyze the addition, removal, and recognition of chemical modifications on RNA molecules, respectively. These modifications play essential roles in post-transcriptional gene regulation and have been linked to aberrant gene expression in various diseases. Several of the enzymes involved, particularly writers and erasers, have emerged as potential therapeutic targets. For instance, inhibition of the methyltransferase METTL3 has been shown to significantly suppress the progression of acute myeloid leukemia (AML) [29]. This review focuses on eight common RNA modifications (m6A, m6Am, m1A, m5C, ac4C, m7G, Ψ, A-to-I editing) and their associated enzymes or proteins (Table 1).

**Table 1:** Common types of RNA modifications and associated enzymes or proteins

Type	Contribution	Writers	Erasers	Readers	Ref.
m6A	mRNA, rRNA, lncRNA, miRNA, circRNA, snRNA, snoRNA	METTL3, METTL14, METTL16, RBM15, RBM15B, VIRMA (KIAA1429), WTAP, ZC3H13, CBLL1 (HAKAI), ZCCHC4, METTL5	ALKBH5, FTO	YTHDC1, YTHDC2, YTHDF1, YTHDF2, YTHDF3, IGF2BP1, IGF2BP2, IGF2BP3, HNRNPC, HNRNPA2B1, HNRNPG, FMR1, RBMX, LRPPRC eIF3, PRRC2A, SND1	[31, 32]
m6Am	mRNA, snRNA	PCIF1 (CAPAM), METTL4	FTO	PCF11	[33, 34]
m1A	mRNA, rRNA, lncRNA, tRNA	TRMT6, TRMT61A, TRMT61B, TRMT10B, TRMT10C, NML (RRP8)	ALKBH1, ALKBH3, ALKBH7, FTO	YTHDF1, YTHDF2, YTHDF3, YTHDC1	[35]
m5C	mRNA, rRNA, lncRNA, tRNA, miRNA, eRNA	NSUN1, NSUN2, NSUN3, NSUN4, NSUN5, NSUN6, NSUN7, DNMT2 NSUN5a/b/c	ALKBH1, TET1, TET2, TET3	ALYREF, YBX1, YBX2, YTHDF2, RAD52, FMRP, SRSF2	[36, 37]
ac4C	mRNA, rRNA, lncRNA, tRNA	NAT10	N/A	N/A	[38]
m7G	mRNA, rRNA, tRNA, miRNA	METTL1/WDR4, WBSCR22/TRMT112, RNMT	N/A	QKI, IGF2BP3, eIF4E, CBC (NCBP1/2)	[39–42]
Ψ	mRNA, rRNA, tRNA, snRNA	DKC1, PUS1, PUS3, PUS7, PUS7L, PUS9, PUS10, RPUSD1, RPUSD2, RPUSD3, RPUSD4, TRUB1, TRUB2	N/A	N/A	[43, 44]
A-to-I editing	mRNA, lncRNA, miRNA	ADAR1, ADAR2, ADAR3	N/A	N/A	[45]

### N6-methyladenosine

m6A refers to the methylation of the sixth nitrogen atom of adenine in RNA molecules, which occurs mainly near stop codons, in 5′- and 3′ -untranslated regions, in long internal exons, and in the shared sequence RRACH (R = A/G and H = A/C/U) [30]. It is the most abundant internal modification in eukaryotic RNA, widely present in mammalian mRNA and various non-coding RNAs [1]. The recognition and binding of m6A modification depend on multiple reader proteins [31, 32], as shown in Table 1.

### N6,2’-O-dimethyladenosine

m6Am is an RNA modification formed by N6-methylation of 2’-O-methyladenosine, occurring primarily near the m7G cap structure of mRNA and snRNA [33]. FTO is the only known demethylase [33]. Additionally, An et al. demonstrated that the transcription terminator PCF11 can function as a specific reader of m6Am [34].

### N1-methyladenosine

m1A is a modification involving the methylation of the N1 position of adenosine. This process is catalyzed by a group of methyltransferases, primarily including TRMT6, TRMT61A, TRMT61B, TRMT10B, TRMT10C, and NML [35].

### 5-methylcytosine

The m5C modification involves the addition of methyl groups to cytosine residues in RNA molecules, forming 5-methylcytosine. This modification plays a crucial role in regulating RNA stability, export and translation. Known m5C readers include eight proteins [36, 37].

### N4-acetylcytosine

The ac4C modification refers to the chemical modification resulting from acetylation at the N4 position of cytosine. Currently, NAT10 is the only known ac4C ‘writer’ [38].

### N7-methylguanosine

m7G is an RNA methylation modification at the N7 position of guanine. It plays a variety of roles in different RNA types, including RNA maturation, nuclear export, and translation [39]. Known readers of m7G include QKI and IGF2BP3 [40, 41]. However, research on m7G-modifying enzymes, particularly the ‘erasers’ remains insufficient [42].

### Ψ

Ψ, the C5-ribosyl isomer of uridine, is a post-transcriptional modification catalyzed by a group of enzymes known as pseudouridine synthases. At present, thirteen pseudouridine synthases are known, including DKC1, PUS1, PUS3, PUS7, PUS7L, PUS9, PUS10, RPUSD1, RPUSD2, RPUSD3, RPUSD4, TRUB1, TRUB2 [43, 44].

### A-to-I editing

A-to-I RNA editing is a common post-transcriptional modification widely present in animal cells, primarily catalyzed by the adenosine deaminase acting on RNA family of enzymes [45].

RNA modifications play essential roles in both coding and non-coding RNA molecules [31, 33, 35, 36, 38, 42, 43, 45]. The biological functions of eight representative RNA modifications are summarized in Table 2.

**Table 2:** Biological functions of eight RNA modifications

Modifications	Target RNA	Biological function	Ref.
m6A	mRNA	Regulates transcription, nuclear export, stability, splicing, translation, and degradation	[31]
	rRNA	Promotes translation	
	lncRNA	Modulates structure and stability	
	miRNA	Facilitates pri-miRNA processing	
	circRNA	Regulates biogenesis, export, translation, and stability	
	snRNA	Affects snRNA-mediated pre-mRNA splicing	
m6Am	mRNA	Enhances stability	[33]
	snRNA	Affects pre-mRNA splicing	
m1A	mRNA	Regulates translation and stability	[35]
	rRNA	Maintains ribosomal integrity	
	tRNA	Stabilizes tRNA structure and influences translation initiation	
m5C	mRNA	Regulates stability, export and translation	[36]
	rRNA	Regulates stability and translation	
	lncRNA	Affects stability	
	tRNA	Regulates stability and translation	
ac4C	mRNA	Enhances stability and translation efficiency	[38]
	rRNA	Regulates rRNA biogenesis	
	lncRNA	Regulates stability	
	tRNA	Improves the fidelity of protein translation	
m7G	mRNA	Regulates stability, transcription, splicing, translation initiation and degradation rate	[42]
	rRNA	Impacts the structural stability and folding of rRNA	
	tRNA	Affects tRNA folding, structural stability, translation initiation and efficiency	
Ψ	mRNA	Regulates splicing and translation	[43]
	rRNA	Improves the conformational stability, controls translational fidelity	
	tRNA	Facilitates tRNA-derived fragments generation, enhances the stability of anticodon pairing	
	snRNA	Influences structure	
A-to-I editing	mRNA	Regulates localization, translation, and stability	[45]
	miRNA	Regulates miRNA maturation and stability	

### RNA modification and diseases

Aberrant RNA modifications are closely associated with the onset and progression of various diseases [32, 33, 35, 37–39, 42, 44–47]. Table 3 summarizes eight RNA modifications (m6A, m6Am, m1A, m5C, ac4C, m7G, Ψ, and A-to-I editing) and their related diseases.

**Table 3:** Eight kinds of RNA modifications and associated diseases

Modifications	Diseases	Ref.
m6A	cancers; neuropsychiatric disorders (such as major depressive disorder and schizophrenia); metabolic diseases (such as type 2 diabetes, non-alcoholic fatty liver disease, and obesity); cardiovascular conditions (such as ischemic heart disease and pulmonary arterial hypertension); immune diseases (such as rheumatoid arthritis, systemic lupus erythematosus, multiple sclerosis and psoriasis)	[31, 32, 46]
m6Am	obesity; gastric cancer, colorectal cancer, glioma; viral infections	[33]
m1A	cancers, including gastrointestinal, hepatocellular, glioma, breast cancer, ovarian cancer, non-small cell lung cancer, colorectal cancer, and prostate cancers	[35]
m5C	genetic disorders; neurological diseases; cancers; autoimmune conditions; infertility; infections	[37]
ac4C	cancers, cardiovascular diseases, metabolic diseases; neurological diseases; infectious diseases; autoimmune diseases	[38]
m7G	cancers; nervous system diseases	[39, 42]
Ψ	cancers; cardiovascular diseases; neurological diseases; metabolic diseases	[44]
A-to-I editing	cancers; neurological diseases	[45, 47]

**Figure 2:**
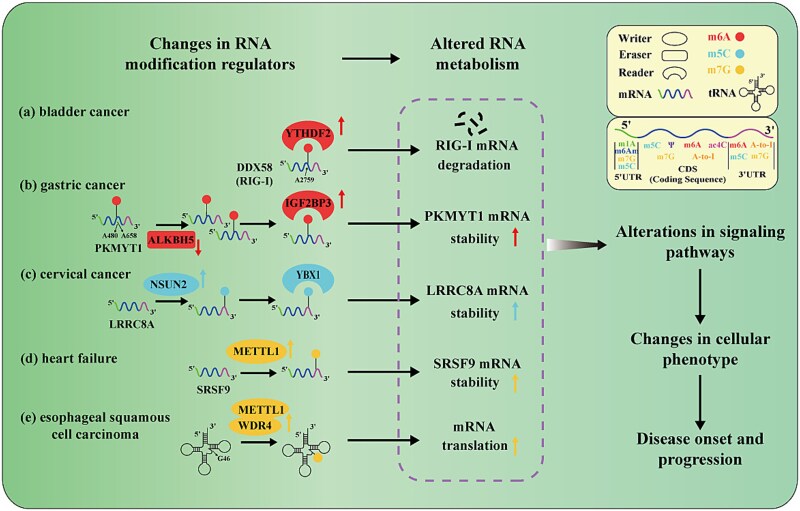
Regulatory mechanisms of RNA modifications in disease occurrence and progression. ‘Writers’ catalyze the addition of modifications to RNA, ‘erasers’ remove these modifications, and ‘readers’ recognize the modified RNA and recruit the appropriate molecular machinery during translation. These modification regulators change the modification state of the target gene RNA, affecting RNA metabolism, thereby altering signaling pathways and cell phenotypes, and ultimately affecting disease onset and progression.

The regulatory mechanisms of RNA modifications in disease onset and progression are illustrated in Fig. 2. Alterations in the activity of RNA-modifying enzymes lead to changes in the modification status of specific RNA molecules, thereby affecting the stability, splicing, or translational efficiency of the target RNA. These molecular changes disrupt the regulation of critical signaling pathways, resulting in aberrant cellular behaviors that ultimately contribute to disease onset and progression [3, 48–51].

In bladder cancer (Fig. 2a), elevated YTHDF2 recognizes m6A-modified DDX58 mRNA at its CDS GGAC motif, promoting its degradation and impairing RIG-I-mediated type I IFN signaling. This leads to decreased apoptosis, enhanced immune evasion, and facilitates the progression from non-muscle-invasive bladder cancer to MIBC [49]. Similarly, in heart failure (Fig. 2d), the upregulation of METTL1 increases m7G modification of SRSF9 mRNA, thereby enhancing its stability and protein expression. Elevated SRSF9 levels promote the alternative splicing of NFATc4 pre-mRNA and the activation of hypertrophic gene expression, thereby activating the NFAT signaling pathway, leading to cardiac hypertrophy and remodeling, and ultimately promoting the occurrence and development of heart failure [50].

RNA modifications regulate key signaling pathways through diverse molecular mechanisms, exerting disease-specific regulatory effects. In addition to the type I interferon and NFAT pathways discussed above, numerous other signaling cascades have been implicated in RNA modification-mediated disease processes. For example, oncogenic pathways such as the MYC, Wnt/β-catenin, p53, and BCL-2 pathways are frequently modulated by m6A-related regulators, influencing tumor progression [52]. Moreover, different RNA modifications may exhibit synergistic effects on the same pathway. For instance, GPX4 transcripts in colorectal adenocarcinoma are co-modified by m6A and m5C, thereby activating the cGAS-STING pathway to maintain redox homeostasis and promote antitumor immunity [53].

Although most studies have focused on mRNA modifications, an increasing number of studies have shown that RNA modifications also play a key regulatory role in non-coding RNAs, including miRNAs, snRNAs, snoRNAs, and lncRNAs, thereby contributing to disease initiation and progression. For instance, the m6A reader HNRNPA2B1 facilitates the maturation of miR-106b-5p in an m6A-dependent manner, leading to SFRP2 suppression and activation of the Wnt/β-catenin pathway, thereby promoting proliferation and migration in lung adenocarcinoma [54]. Pseudouridylation mediated by snoRNAs and their associated enzymes has been implicated in tumor invasiveness; e.g. SNORA23 promotes rRNA pseudouridylation and is associated with increased invasiveness in pancreatic ductal adenocarcinoma [44]. In lncRNA, METTL3-mediated m6A modification of LINC00958 enhances its stability and facilitates hepatocellular carcinoma progression via the LINC00958/miR-3619-5p/HDGF axis [55]. These regulatory mechanisms highlight the clinical relevance of RNA modifications in ncRNAs and their potential as biomarkers.

## Biomarker discovery based on machine learning

**Figure 3:**
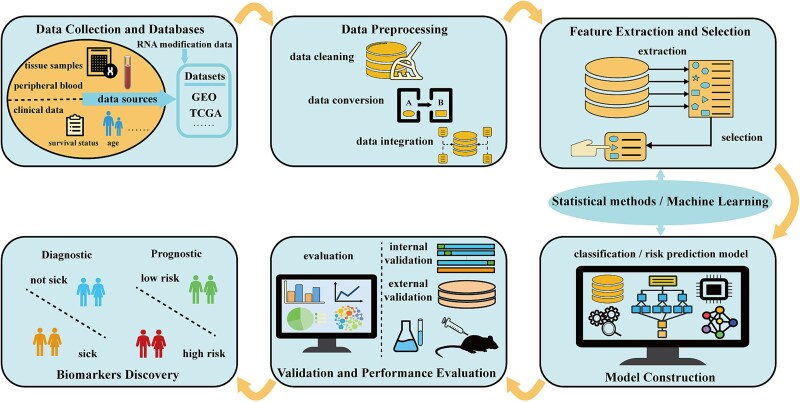
Workflow for using machine learning to identify RNA modification related genes as diagnostic or prognostic biomarkers for diseases.

The workflow of RNA modification–related disease diagnosis or prognostic biomarker identification based on machine learning is shown in Fig. 3.

In studies of diagnostic and prognostic biomarkers, datasets are primarily sourced from repositories such as GEO, TCGA, and Genotype-Tissue Expression (GTEx). Prognostic analyses also require clinical information, such as patient survival and disease stage. The RNA modification regulators used for analysis are typically derived from previous studies and literature. Data preprocessing involves batch effect correction, normalization of expression data, and matching with clinical data. Subsequently, statistical methods and machine learning algorithms, either individually or in combination, are applied for feature extraction and selection, as well as to construct diagnostic or prognostic models. For example, in diagnostic modeling, differentially expressed genes (such as |logFC| > 1, *P* < .05) can be identified using the limma package, and feature subsets were determined by ranking features based on variable importance scores of RF [56]. For prognostic modeling, univariate Cox regression can be used for initial screening (such as *P* < .05), followed by LASSO regression to compress redundant variables and retain predictors with nonzero coefficients [57]. Model performance is assessed using established metrics such as the AUC and the C-index.

Where feasible, experimental approaches, including qRT-PCR for verifying gene expression trends, western blot for assessing protein levels, and animal-based or cell-based functional assays, are used to further validate the findings. In addition, clustering analysis based on RNA modification regulators can be performed to categorize disease samples into distinct groups. Differences among these groups can then be examined in terms of gene expression, survival outcomes, functional pathways, immune cell infiltration, and drug sensitivity. This analysis helps to uncover potential biological heterogeneity and provides a molecular basis for precision medicine.

### Diagnostic biomarkers

Diagnostic markers are biomarkers used to detect the presence or absence of a disease and to identify its subtypes. Machine learning techniques have been employed to identify diagnostic biomarkers across various disease categories, including cardiovascular and cerebrovascular diseases, infectious diseases, immune diseases, and bone and joint diseases (Tables 4–7). The screened biomarkers exhibit significantly aberrant expression in these diseases. These markers emerge throughout both the early and progressive stages, serving as dynamic targets for diagnosis, and treatment. The following section reviews the application of machine learning approaches in the identification of diagnostic biomarkers across four selected disease categories.

**Table 4:** Research on RNA modifications combined with machine learning algorithms for cardiovascular and cerebrovascular diseases diagnostic biomarkers

RNA Type	Disease	Sample Size and Source	Diagnostic Biomarkers	Models and Approaches	Results	Ref.
m7G	IS	89 IS patient samples and 43 control samples; GEO database	EIF3D, CYFIP2, NCBP2, DCPS, NUDT1	RF, SVM, screen key genes	AUC = 0.967; RF (AUC = 1) is better than SVM (AUC = 0.946)	[58]
m6A	ICM	118 ICM patients and 22 healthy subjects (training set), 136 ICM patients and 95 healthy subjects (testing set); GEO database	WTAP, ZCH3H13, YTHDC1, FMR1, FTO, RBM15, YTHDF3	RF, screen key genes	AUC = 0.934 (training set), AUC = 0.891 (testing set)	[59]
m6A	AS	35 healthy individuals and 69 AS patients, scRNA-seq data of 3 carotid artery plaques and 3 adjacent healthy tissues; GEO database	METTL5	SVM-RFE, RF, screen key genes	AUC = 0.850	[60]
m7G	PAH	26 PAH patients and 13 normal (training set), 15 PAH patients and 11 normal (testing set); GEO database	CYFIP1, EIF4E, IFIT5	LASSO, RF and SVM-RFE, screen key genes	AUC = 0.956 (training set), AUC = 0.721 (validation set)	[61]

Note: IS, Ischemic stroke; ICM, Ischaemic cardiomyopathy; AS, atherosclerosis; PAH, Pulmonary arterial hypertension.

**Table 5:** Research on RNA modifications combined with machine learning algorithms for infectious diseases diagnostic biomarkers

RNA Type	Disease	Sample Size and Source	Diagnostic Biomarkers	Models and Approaches	Results	Ref.
m5C	Sepsis	47 normal samples and 184 sepsis samples (test set), 54 normal samples and 514 sepsis samples (validation set); GEO database	DNMT1, TP53, TLR8	DT, RF, XGBoost, screen genes	the AUC of DNMT1, TP53, and TLR8 was 0.979, 0.967, 0.944	[62]
m1A, m5C, m6Am, m7G and Ψ	Sepsis	839 sepsis patient samples and 87 control samples; GEO database. 39 adult patients and 13 healthy controls (recruited)	NSUN7, NOP2, PUS1, PUS3, FTO	RF, GLM, SVM and XGBoost, screen key genes	the AUC of NSUN7, NOP2, PUS1, PUS3 and FTO was 0.828, 0.707, 0.846, 0.834, 0.976; SVM is superior to the other three models (AUC = 0.998)	[26]
m7G	CHB	128 liver samples and 124 HBV-related liver fibrosis samples; GEO database. 30 healthy people and 39 CHB peripheral blood samples (clinical samples).	LARP1, GEMIN5	SVM-RFE, RF, screen key genes	AUC = 0.985 (LARP1) and AUC = 0.964 (GEMIN5); validation set: AUC = 0.897 (LARP1) and AUC = 0.876 (GEMIN5)	[63]
m6A	COVID-19	100 COVID-19 patients and 26 non-COVID-19 patients; GEO database	RBM15B, ELAVL1, RBM15, FMR1, IGFBP3, METTL3, VIRMA, HNRNPA2B1	RF, SVM, identify candidate m6A regulators	RF (AUC = 1) is better than SVM (AUC = 0.975); calibration curves, verify the accuracy	[64]

Note: COVID-19, coronavirus disease 2019; CHB, Chronic hepatitis B.

**Table 6:** Research on RNA modifications combined with machine learning algorithms for immune diseases diagnostic biomarkers

RNA Type	Disease	Sample Size and Source	Diagnostic Biomarkers	Models and Approaches	Results	Ref.
m6A	Psoriasis	170 human skin tissue samples (training dataset), 180 human skin tissue samples (validation dataset); GEO database	FTO, IGF2BP2, METTL3, YTHDC1, ZC3H13, HNRNPC, IGF2BP3, LRPPRC, YTHDC2, HNRNPA2B1	LR, LASSO, select the feature genes; SVM, construct the diagnostic model	AUC = 0.974 (training set), AUC = 0.730 (validation set)	[27]
m6A	childhood asthma	40 non-asthmatic and 65 asthmatic patients; GEO database	FMR1, KIAA1429, WTAP, YTHDC2, ZC3H13	RF, SVM, screen candidate m6A regulators	RF (AUC = 0.87) is better than SVM (AUC = 0.84)	[15]

**Table 7:** Research on RNA modifications combined with machine learning algorithms for bone and joint diseases diagnostic biomarkers

RNA Type	Disease	Sample Size and Source	Diagnostic Biomarkers	Models and Approaches	Results	Ref.
m6A	OA	30 OA patients and 29 healthy control population; GEO database	YTHDF2	RF, SVM, screen key genes	AUC = 0.875, 95% CI: 0.778–0.952; RF (AUC = 1) is better than SVM (AUC = 0.899)	[65]
A-to-I, APA, m5C, m6A, m7G, mcm5s2U, Nm and Ψ	OA	contain 30 OA and 10 normal sub-chondral samples; GEO database	WDR4, CFI	RF, SVM, screen key genes	AUC = 0.925, 95% CI: 0.825–1.000 (WDR4); AUC = 0.950, 95% CI: 0.882–0.995 (CF1); the residual of RF is smaller than that of SVM	[56]
m6A	osteoporosis	40 high and 40 low hip BMD monocyte samples (training cohort), 14 high and 12 low hip BMD monocyte samples (validation cohort); GEO database	FTO, YTHDF2, CBLL1	LASSO, SVM-RFE, screen hub genes	Training cohort AUC = 0.683; validation cohort AUC = 0.732	[66]

Note: OA, osteoarthritis; CI, confidence interval.

Cardiovascular and cerebrovascular diseases are often associated with abnormalities in the blood circulation system, impaired heart function, or insufficient blood supply to the brain, and they have a high mortality rate in the world. Studies have shown that these diseases are associated with dynamic changes in RNA modifications such as m6A and m7G [58–61] (Table 4).

Tian et al. [58] analyzed two mRNA expression datasets related to ischemic stroke (IS) to identify m7G differentially expressed genes (DEGs) in samples of ischemic stroke patients and healthy controls, and then further identified key m7G regulatory genes in IS through RF algorithm. Subsequent validation using a middle cerebral artery occlusion model and qPCR confirmed that the identified m7G-regulated genes play essential regulatory roles in IS. The resulting biomarker panel, including EIF3D, CYFIP2, NCBP2, DCPS, and NUDT1, achieved an AUC of 0.967 for IS prediction. In ischemic cardiomyopathy (ICM), Zheng et al. [59] employed an RF model to identify seven key m6A regulators, namely genes encoding m6A-related proteins, and constructed a diagnostic nomogram based on their expression levels. Additionally, clustering algorithms and PCA were applied to identify two distinct m6A modification patterns, thereby stratifying ICM patients into subgroups with differing immune regulatory mechanisms, and comparing the expression changes of biomarkers in different subgroups. Similarly, Wang et al. [61] used the intersection of the feature selection results of three machine learning methods, namely LASSO, RF, and support Vector Machine recursive feature elimination (SVM-RFE), as the diagnostic feature gene for pulmonary arterial hypertension (PAH). Additionally, the association between the selected signature genes and the drugs in DSigDB database was analyzed, and the utility of these drugs was evaluated based on p-values and other relevant metrics, providing new ideas for the treatment of PAH.

Infectious diseases are caused by pathogens, including bacteria, viruses, fungi, and parasites, which invade the host and trigger clinical symptoms, such as sepsis, chronic hepatitis B and COVID-19 [26, 62–64]. Lin et al. [62] integrated two sepsis related datasets and corrected for batch effects, applied DT, RF, and XGBoost to identify key genes of sepsis m5C modification. The common genes identified by all three methods were DNMT1, TP53, and TLR8, with diagnostic AUC values of 0.979, 0.967, and 0.944, respectively. Zhang et al. [26] analyzed five classes of RNA modification-related genes (m1A, m5C, m6Am, m7G, and Ψ) to identify novel diagnostic biomarkers for sepsis. Unsupervised clustering revealed distinct RNA modification subtypes, followed by CIBERSORT, WGCNA, GO, and KEGG analyses to characterize immune infiltration patterns and biological functions associated with each subtype.

Immune diseases are disorders caused by abnormalities or dysfunctions in the immune system. Liu et al. [27] explored the diagnostic potential of m6A regulators in psoriasis and identified 10 DEGs associated with m6A using logistic regression and the LASSO algorithm, and then constructed a diagnostic classifier using SVM. Dai et al. [15] compared the effects of RF and SVM models in screening characteristic genes and found that the RF model (AUC = 0.87) had higher accuracy than SVM (AUC = 0.84). Eventually, RF was selected to screen five candidate m6A regulators, and cluster analysis was conducted to divide children with asthma into two different m6A modification patterns. These patterns effectively distinguished allergic from non-allergic asthma and guide the subsequent treatment.

Bone and joint diseases, which affect bones, joints, can cause pain, swelling, stiffness, and dysfunction, significantly impairing patients’ quality of life, such as osteoarthritis (OA) and osteoporosis (OP). Bian et al. [65] compared the effects of RF and SVM in the feature selection of m6A regulatory factors for OA synovitis and found that the AUC of RF was greater than that of SVM, indicating that the accuracy of the RF model was higher than that of SVM. Chen et al. [56] studied the specific effects of eight RNA modifiers in OA and their association with immune infiltration. Among the identified biomarkers, WDR4 and CFI demonstrated diagnostic performance, with AUC values of 0.925 and 0.950, respectively. Bai et al. [66] developed both the LASSO regression and the SVM-RFE model to screen candidate m6A regulators for predicting OP from 13 differentially expressed m6A regulators. By intersecting results from both models, they selected FTO, YTHDF2, and CBLL1 as potential biomarkers.

### Prognostic biomarkers

Prognostic markers are biomarkers that predict clinical outcomes, including disease recurrence, progression, and patient survival, in individuals with confirmed diagnoses. RNA modifications regulate the expression of oncogenes and tumor suppressor genes by modulating processes such as transcription, RNA splicing, mRNA stability, and translation, thereby influencing tumor initiation and progression.

In general, diseases with a complex course and high heterogeneity require prognostic evaluation, such as malignant tumors and chronic inflammatory diseases. Prognostic models based on molecular subtypes can evaluate the survival of different subgroups of patients, guide the development of personalized treatment plans, and optimize the priority allocation of medical resources through risk stratification. For instance, lung adenocarcinoma (LADC) patients stratified by median risk score were divided into high-risk and low-risk groups, with the high-risk group exhibiting significantly shorter survival times, which guides targeted therapy strategies. Moreover, analysis using the Tumor Immune Dysfunction and Exclusion algorithm indicated that immune checkpoint blockade therapy might be beneficial for high-risk LADC patients, whose tumor tissues have higher expression levels of PD-L1 and PD-L2 [57]. Relevant studies of tumor prognostic biomarkers related to RNA modification are summarized in Table 8.

**Table 8:** Research on RNA modifications combined with machine learning algorithms for disease prognostic biomarkers

RNA Type	Disease	Sample Size and Source	Prognostic Biomarkers	Models and Approaches	Results	Ref.
m1A	BRCA	1069 BRCA samples and 114 normal samples, TCGA; 115 BRCA samples, GEO	MEOX1, COL17A1, FREM1, CD1C, TNN, SLIT3	univariate Cox, LASSO Cox	AUC = 0.763 (1 year), AUC = 0.658 (3 years), AUC = 0.646 (5 years)	[67]
m6A	STAD	375 STAD tumor samples, 361 normal tissues samples; TCGA, GTEx	IGF2BP1, RBM15, FTO, ALKBH5	Univariate and Multivariate Cox Regression, LASSO	1 year (AUC = 0.743), 3 years (AUC = 0.743), 5 years (AUC = 0.874)	[68]
m6A	RC	95 RC patients and 10 normal adjacent tissues, TCGA; 203 RC tumor tissue samples, GEO	RBMX, LRPPRC	Univariate and Multivariate Cox Regression, LASSO	*P* = .022 (YTHDC2); *P* = .016 (METTL14)	[69]
m6A	LADC	398 LADC patients samples, TCGA; 468 LADC patients samples, GEO;	IGF2BP1, IGF2BP2, HNRNPA2B1, METTL3, HNRNPC	univariate Cox, LASSO Cox	3 years (AUC = 0.684), 5 years (AUC = 0.646), C-index = 0.71	[57]
m6A	MM	859 MM samples and 337 normal samples; GTEx	HNRNPA2B1, KIAA1429	LASSO Cox regression, PCA	5-year (AUC = 0.792)	[77]
m6A	Colorectal cancer	175 samples (training set), 3 validation sets; GEO	RBM15B, FTO, IGF2BP2, ZCCHC4, KIAA1429	multivariate Cox regression, LASSO, clustering, PCA	1 year (AUC = 0.64), 5 years (AUC = 0.67)	[72]
m6A	Glioma	665 glioma patient samples, TCGA; 420 LGG patients and 237 GBM patients samples, CGGA	TAGLN2, PDPN, TIMP1, EMP3	PCA, LASSO Cox	AUC = 0.8 (TCGA), AUC = 0.72 (CGGA)	[73]
m6A	READ	88 READ samples; TCGA	ADAMTSl1, CSMD2, FAM13C, FAM184A, KLHL4, OLFML2B, PDZD4, SEC14L5, SETBP1, TMEM132B	PCA, Univariate Cox Regression, SVM	AUC = 0.8630 (3 years), AUC = 0.8721 (prognosis status)	[70]
m6A	HNSCC	506 HNSCC Samples; TCGA	ALKBH5, YTHDC2	Univariate and Multivariate Cox Regression, Random forest, neural network model	YTHDC2 was selected as the most prognostically important locus of the 10 m6A regulatory genes in HNSCC	[71]
m6A	PC	176 PC patients; TCGA, GEO	IGF2BP2	PCA, univariate Cox regression, LASSO Cox	HR = 2.19, higher IGF2BP2 contributed to lower survival rate	[28]
m7G	HCC	365 HCC patients samples; TCGA	NCBP2	LASSO, PCA, t-SNE, Univariate and Multivariate Cox Regression	AUC = 0.682 (1 year), AUC = 0.638 (3 years), AUC = 0.601 (5 years)	[74]
m7G	AML	150 AML samples, TCGA; 104 AML samples, GEO	CBR1, CCDC102A, LGALS1, RD3L, SLC29A2, TWIST1	PCA, univariate Cox, LASSO	AUC = 0.871 (1 year), AUC = 0.874 (3 years), AUC = 0.951 (5 years)	[75]
m7G	Sarcoma	260 sarcoma instances and 2 non-cancerous tissue samples, TCGA; 3 GEO datasets	EIF4A1, EIF4G3, NCBP1, WDR4	univariate Cox, LASSO Cox	AUC = 0.724 (1 year), AUC = 0.638 (3 years), AUC = 0.718 (5 years)	[76]

Note: BRCA, breast carcinoma; STAD, gastric adenocarcinoma; RC, Rectal cancer; LADC, lung adenocarcinoma; MM, multiple myeloma; READ, rectum adenocarcinoma; HNSCC, head and neck squamous cell carcinoma; PC, pancreatic carcinoma; HCC, hepatocellular carcinoma; AML, acute myeloid leukemia.

Li et al. [67] collected RNA-Seq, copy number variation, single nucleotide variation, and clinical data for breast cancer samples from TCGA. Using univariate Cox regression analysis and the LASSO algorithm, they identified six m1A modification associated genes as prognostic biomarkers and constructed a risk model, which was externally validated using GEO dataset with consistent results. Zhao et al. [68] used univariate Cox regression to screen genes related to the survival of gastric adenocarcinoma (STAD), and a LASSO-based prognostic model was developed, and based on the median risk score, STAD patients were stratified into low-risk and high-risk subgroups. Chen et al. [69] applied LASSO regression to construct a prognostic model for rectal cancer (RC) patients, revealing that low expression of YTHDC2 and METTL14 was significantly associated with poorer overall survival. Huang et al. [70] used a SVM model to predict 3-year survival rates, prognostic status, and pathological stage of RC based on risk scores derived from survival-associated signature genes, achieving AUCs of 0.863, 0.8721, and 0.8752, respectively. Zhou et al. [71] utilized RF and neural network models to rank the expression of 10 m6A regulators in head and neck squamous cell carcinoma tumor and normal tissues. Among these, YTHDC2 emerged as the most significant prognostic biomarker. In studies of tumor prognostic markers, LASSO is frequently combined with Cox proportional hazards regression to efficiently select predictive variables and assess survival outcomes [28, 57, 68, 72–77].

By integrating machine learning algorithms with statistical and bioinformatics approaches, robust prognostic models can be developed to assess patient risk scores, thereby facilitating personalized treatment strategies.

## Discussion

### Disease data sets and data sources

In the reviewed literature, studies on diagnostic and prognostic biomarkers predominantly utilize datasets from the GEO and TCGA databases, which are generated using various chip platforms and detection technologies, e.g. Affymetrix, Agilent, Illumina microarray chip platforms, variations among these platforms can affect result accuracy and marker screening [78]. Thus, integrating datasets from multiple platforms can enhance the reliability of RNA modification related biomarkers identification [26]. Gene chip technology is well-established, and machine learning can rapidly identify candidate biomarkers from the structured data matrices produced by microarrays. Next-generation sequencing (NGS) has been employed to characterize disease transcriptome via RNA-seq, thereby providing information on gene expression. However, its short reads can limit the analysis of complex transcripts. In contrast, third-generation sequencing, with its long-read capabilities, can resolve intricate transcript structures and directly detect epigenetic modifications, although its sequencing error rate is higher than that of NGS. Furthermore, gene chip technology is generally less expensive than NGS, NGS less expensive than third-generation sequencing. In the reviewed literature, most studies utilized microarray data, while some employed processed RNA-seq gene expression profiles obtained from NGS.

Many studies validate their findings by employing data from different platforms, e.g. using the TCGA dataset as the training set and the GEO dataset for validation, to ensure robustness and external consistency [67, 76]. Furthermore, sample size plays a crucial role in study stability, as small clinical cohorts may lead to model overfitting. In addition to that, studies have demonstrated crosstalk among different RNA modification types. Consequently, instead of relying on a single modification, combining data on multiple RNA modification types can enhance the identification of disease biomarkers [69].

### Applications of machine learning

Among the reviewed literature, the most commonly used machine learning methods were supervised and unsupervised learning.

Various supervised learning algorithms, including RF, DT, SVM, and XGBoost, are widely employed to screen disease key genes from large datasets, construct diagnostic or prognostic models [26, 27, 62]. In comparative analyses of feature selection methods across reviewed literature, RF algorithm demonstrated superior robustness and accuracy. For instance, in the context of COVID-19, the RF model achieved an AUC of 1.0 following 10-fold cross-validation, outperforming the SVM model, which yielded an AUC of 0.975 [64]. Similarly, in a tuberculosis diagnostic study based on m6A regulatory genes, four machine learning algorithms, RF, SVM, XGB, and GLM, were compared for their ability to identify relevant regulators [79]. The RF model had the smallest residuals, indicating better overall performance. Its AUC reached 1.0, higher than those of SVM (0.926), XGB (0.992), and GLM (0.816), with ROC analysis further confirming the robustness of RF. Due to its ensemble decision-tree structure, strong robustness, and ability to rank feature importance, RF is widely adopted for feature selection in RNA modification-based biomarker studies. Unsupervised learning algorithms, such as clustering and PCA, can be used to distinguish between different RNA modification patterns and disease subtypes [28, 59, 77]. To improve the accuracy of biomarker screening, several studies compare and integrate results from multiple machine learning models to determine the optimal algorithm or combination thereof. For instance, the intersection of feature genes identified by multiple machine learning approaches can also be employed to define key regulatory genes [61].

Additionally, deep learning models, like artificial neural networks, can also be applied to disease biomarker mining [71]. However, deep learning is rarely applied in this field due to several challenges. First, available datasets are typically small, often comprising only tens to hundreds of samples, making conventional machine learning algorithms more suitable. These algorithms reduce the risk of overfitting and require less training time, whereas deep learning models generally demand large-scale data and incur high computational costs. Second is the lack of interpretability. Traditional machine learning methods can provide clear insights into variable contributions, the inherent ‘black box’ nature of deep learning limits its transparency and hampers clinical translation. Deep learning is more suitable for the identification of biomarkers by multimodal data integration and the prediction of RNA modification sites, which can improve the prediction performance. Therefore, integrating RNA modification data with other omics datasets, introducing attention mechanism, SHAP and other methods to enhance the interpretability, may further facilitate the application of deep learning in this field.

### RNA modification and liquid biopsy

Liquid biopsy technology also has great potential in identifying disease biomarkers associated with RNA modifications. Compared to traditional tissue biopsy, liquid biopsy offers advantages such as convenient sampling, high safety, minimal discomfort, and real-time monitoring of disease progression [80]. Peripheral blood, one of the most common sample types in liquid biopsy, can extract various biomarkers for analysis. Among them, the detection of RNA modification levels can serve as a valuable biomarker for non-invasive disease diagnosis. For instance, the level of m5C modification in peripheral blood immune cells of colorectal cancer patients increases with cancer progression and metastasis but significantly decreases post-treatment. The AUC for m5C modification level in diagnosing colorectal cancer was 0.888, outperforming conventional serum biomarkers such as CEA (0.739), CA19–9 (0.669), and CA125 (0.629) [81]. Similarly, combining peripheral blood m6A modification levels with METTL14 and FTO regulatory genes achieved an AUC of 0.929 for breast cancer diagnosis, which was also better than the biomarkers CEA (0.599) and CA153 (0.572). [82]. These studies consistently indicate that specific RNA modification profiles outperform traditional serum biomarkers, highlighting their potential for clinical translation.

### RNA modification related biomarkers and cross-disease research

Although RNA modification-related biomarkers have shown diagnostic and prognostic value in specific disease contexts, their generalizability across diverse disease types remains largely unexplored. Current efforts have primarily focused on pan-cancer analyses. For instance, studies have examined the expression and mutation of 26 RNA modification writers (RMWs) across 32 cancer types and found that most RMWs are highly expressed in tumor tissues and associated with poor patient prognosis, indicating that these biomarkers have certain commonalities [83]; evaluated the diagnostic and prognostic potential of the m6A reader YTHDC2 in pan-cancer settings [84]; conducted a pan-cancer analysis of 26 m7G regulators across 17 tumor types using bioinformatics approaches, the m7Gscore was found to be strongly correlated with prognosis in most cancers, highlighting the potential of m7G regulators as prognostic biomarkers [85]. Future studies should systematically compare the expression patterns, regulatory mechanisms, and predictive performance of RNA modification factors across multiple diseases, to identify both shared and disease-specific biomarker. Integrating multi-disease datasets with machine learning approaches will enable comprehensive analyses to assess the cross-disease robustness of these biomarkers, thereby facilitating the transition from disease-specific findings to clinically applicable, broadly useful diagnostic tools.

### Biomarkers related to RNA modifications in ncRNAs

RNA modifications are widely distributed across both coding and non-coding RNAs; however, their biological functions and clinical significance are significantly different between these two RNA classes. In mRNAs, RNA modifications directly influence protein expression, facilitating the identification of their associations with disease phenotypes and enabling more straightforward detection. In contrast, in ncRNAs, modifications primarily modulate RNA structure, processing, and interactions, thereby exerting indirect effects on gene regulatory networks.

While this review focuses on machine learning-based discovery of mRNA-related prognostic biomarkers in RNA modification-associated diseases, some studies highlight that ncRNAs associated with RNA modifications may also serve as potential prognostic biomarkers in various diseases [86, 87]. For example, m7G-related lncRNAs have the potential as prognostic biomarkers of esophageal squamous cell carcinoma [88].

## Limitations and challenges of current research

Although great progress has been made in integrating RNA modification biology with machine learning to identify diagnostic and prognostic biomarkers, several critical challenges remain.

First, many studies rely on small sample sizes or single-cohort data, increasing the risk of overfitting and limiting external validation. Second, biological and technical variability across datasets, such as tissue types, batch effects and sequencing platforms, can introduce noise and confounding factors, thereby affecting the discovery of accurate biomarkers. Third, biological heterogeneity among patients, including differences in tissue origin, disease stage, and comorbidities, adds complexity to biomarker discovery and limits the generalizability of predictive models. Fourth, current RNA modification detection methods, such as MeRIP-seq, suffer from limited resolution, batch effects, and technical biases, resulting in false positives or negatives and reduced reproducibility. In addition, tracking the dynamic regulation of RNA modification remains challenging. Finally, translating these findings into clinical practice is difficult. Most studies lack large-scale prospective validation, which may lead to overestimation of biomarker performance. Extensive clinical trials are necessary to confirm the diagnostic and prognostic value of these markers before their clinical application. In summary, there is great potential in mining new diagnostic and prognostic biomarkers based on RNA modification–related factors, but there are still limitations and challenges that need to be overcome.

## Conclusions and future directions

This article reviews eight common RNA modifications implicated in disease, examines the mechanisms by which these modifications contribute to disease development, and discusses methods for identifying biomarkers associated with RNA modification. Special emphasis is placed on the application of machine learning techniques for discovering diagnostic and prognostic markers, offering new strategies for precision diagnosis, disease surveillance, and drug development.

In the future, integrating multi-omics data, such as various RNA modifications and biopsy images, and employing advanced deep learning algorithms may facilitate the identification of multimodal biomarkers and enable a comprehensive analysis of the RNA modification regulatory network. In addition, future studies should explore the commonalities and differences of RNA-modified biomarkers in different disease types to evaluate their generalization ability and potential clinical application value. Moreover, advances in sequencing technologies and improvements in analytical algorithms have enhanced sequencing speed and enabled more precise detection and analysis of RNA modifications, thereby facilitating in-depth investigations into the complex relationship between RNA modifications and gene expression regulation. For example, nanopore sequencing, a rapid and real-time sequencing technology, enables efficient tracking of RNA modification dynamics during disease progression. This capability holds great promise for early disease detection, dynamic monitoring of disease development, and evaluation of therapeutic efficacy. As sequencing costs continue to decline and portable devices become more widely available, research on RNA modifications is expected to accelerate its translation into clinical applications.

**Key Points**

- This review introduces eight common RNA modifications related to diseases and the regulatory mechanisms of RNA modifications in diseases. To clarify the principle of RNA modification regulated genes as disease biomarkers.
- This article introduces the latest progress of using machine learning to identify disease-related RNA modification related gene markers, including diagnosis and prognosis evaluation. The overall workflow of biomarker screening was analyzed, and the selection of machine learning methods, feature selection strategies, and challenges in research were discussed.
- Although significant progress has been made in the mining of gene markers related to RNA modification, a large number of clinical trials are still needed to verify their effectiveness in clinical applications. In the future, the deep intersection of AI algorithms, multimodal and epigenetic research is expected to promote further research on RNA modification in disease diagnosis and prognosis.

## Supplementary Material

Supplementary_materials_bbaf361

## Data Availability

Data supporting this study are included in the article, and no new data were generated in this study.
