# Supplementary material for: Research on RNA modification in disease diagnosis and prognostic biomarkers: current status and challenges
Source: Brief Bioinform. 2025 Jul 23;26(4):bbaf361. doi: 10.1093/bib/bbaf361 (PMC12284764; doi:10.1093/bib/bbaf361)
Supplement: Supplementary_materials_bbaf361 [file supplementary_materials_bbaf361.doc]

## Material 1. Abbreviations

| Abbreviation | Full name |
| --- | --- |
| ncRNA | non-coding RNA |
| RMPs | RNA-Modifying Proteins |
| ANOVA | analysis of variance |
| RF | Random Forest |
| SVM | Support Vector Machine |
| DT | Decision Tree |
| XGBoost | eXtreme Gradient Boosting |
| LASSO | Least Absolute Shrinkage and Selection Operator |
| PCA | principal component analysis |
| t-SNE | t-Distributed Stochastic Neighbor Embedding |
| AI | artificial intelligence |
| ROC | receiver operating characteristic |
| AUC | area under the ROC curve |
| TPR | true positive rate |
| FPR | false positive rate |
| FN | false negative |
| FP | false positive |
| C-index | concordance index |
| HR | hazard ratio |
| qRT-PCR | quantitative real-time polymerase chain reaction |
| GEO | Gene Expression Omnibus |
| TCGA | The Cancer Genome Atlas |
| GO | Gene Ontology |
| KEGG | Kyoto Encyclopedia of Genes and Genomes |
| WGCNA | weighted gene co-expression network analysis |
| m6A | N6-methyladenosine |
| m6Am | N6,2 '-O-dimethyladenosine |
| m1A | N1-methyladenosine |
| m5C | 5-methylcytosine |
| ac4C | N4-acetylcytosine |
| m7G | N7-methylguanosine |
| Ψ | Pseudouridine |
| A-to-I | adenosine-to-inosine |
| APA | alternative polyadenylation |
| Nm | 2'-O-methylation |
| mRNA | messenger RNA |
| rRNA | ribosomal RNA |
| lncRNA | long non-coding RNA |
| tRNA | transfer RNA |
| miRNA | microRNA |
| circRNA | circular RNA |
| snRNA | small nuclear RNA |
| snoRNA | small nucleolar RNA |
| eRNA | enhancer RNA |
| AML | acute myeloid leukemia |
| NMIBC | non-muscle-invasive bladder cancer |
| GTEx | Genotype-Tissue Expression |
| IS | ischemic stroke |
| DEGs | differentially expressed genes |
| ICM | ischaemic cardiomyopathy |
| AS | atherosclerosis |
| SVM-RFE | support vector machine recursive feature elimination |
| PAH | pulmonary arterial hypertension |
| CHB | chronic hepatitis B |
| COVID-19 | coronavirus disease 2019 |
| OA | osteoarthritis |
| OP | osteoporosis |
| STAD | gastric adenocarcinoma |
| RC | rectal cancer |
| OS | overall survival |
| LADC | lung adenocarcinoma |
| MM | multiple myeloma |
| READ | rectum adenocarcinoma |
| HNSCC | head and neck squamous cell carcinoma |
| PC | pancreatic carcinoma |
| HCC | hepatocellular carcinoma |
| NGS | next-generation sequencing |
| RMWs | RNA modification writers |

## Material 2. Introduction to machine algorithm

| Random forest is an ensemble learning algorithm based on decision tree. By constructing multiple trees and aggregating their predictions, the algorithm enhances both the model’s generalization ability and robustness. It employs bootstrap sampling to randomly extract multiple subsets from the original dataset. For each subset, an independent decision tree is constructed. When splitting at each node, a subset of features are randomly selected as candidates and the optimal feature is selected for splitting. In classification tasks, the final prediction is determined by majority voting among the trees, while in regression tasks, it is calculated as the average of all individual tree predictions. |
| --- |
| Support vector machine (SVM) classify data by identifying the maximum margin hyperplane that optimally separates two classes. Given a labeled training dataset $X=\left\{(x_{i},y_{i})\right\}_{i=1}^{n},\text{ }x_{i}\in R^{d},y_{i}\in(-1,+1)$, where $x_{i}$ represents the feature vector and $y_{i}$ is its corresponding class label, the optimal hyperplane is defined by the equation $wx^{T}+b=0$, where $w$ is the weight vector and $b$ is the bias term. To ensure correct classification with a margin, every sample must satisfy the condition $\left\{\begin{matrix} wx_{i}^{T}+b\geq1,\text{ }y_{i}=1 \\ wx_{i}^{T}+b\leq-1,\text{ }y_{i}=-1 \end{matrix}\right.$. For linearly separable data, the SVM optimization problem seeks to minimize $\frac{1}{2}\left\Vert w\right\Vert^{2}$ subject to these constraints, $\min_{w,b} \frac{1}{2}\left\Vert w\right\Vert^{2}\text{ }s.t.\text{ }y_{i}(w^{T}\cdot x_{i}+b)\geq1,\forall i$, forming a convex quadratic programming problem. In cases where the data is not linearly separable, a kernel function—such as the radial basis function ($K_{RBF}(x,y)=\exp(-\gamma\left\Vert x-y\right\Vert^{2})$)—is used to project the data into a higher-dimensional space where a linear separation becomes feasible. |
| Decision tree algorithms operate by recursively selecting the optimal feature to partition the dataset until a predetermined stopping criterion is met. In classification tasks, the prediction is made by traversing the tree from the root to a leaf node, where the final output is typically determined by the majority class label of the samples in that leaf. By prioritizing the most informative features for splitting, decision trees not only enhance predictive accuracy but also reduce model complexity by effectively disregarding irrelevant features. Common decision tree algorithms, such as ID3, C4.5, and CART, primarily differ in their feature selection criteria and tree-building strategies. The construction of a decision tree generally involves three key steps: (1) feature selection, wherein the importance of each feature is evaluated using metrics such as information gain or Gini impurity to choose the best splitting attribute; (2) recursive partitioning, where the dataset is split into subsets based on the chosen feature, thereby forming the branches of the tree; and (3) pruning, which is applied to eliminate branches that do not contribute significantly to the model’s performance, thereby preventing overfitting. Pruning techniques are typically classified as either pre-pruning or post-pruning. |
| eXtreme Gradient Boosting (XGBoost) is an optimized implementation of gradient boosting decision trees (GBDT). The XGBoost library is a highly optimized, distributed gradient boosting framework that is efficient, flexible, and lightweight. While it is based on the principles of GBDT, XGBoost incorporates several key enhancements. For example, it employs a second-order Taylor expansion to approximate the loss function, thereby improving the calculation accuracy. Moreover, a regularization term is incorporated into the objective function to control model complexity and mitigate overfitting. Moreover, a regularization term is incorporated into the objective function to control model complexity and mitigate overfitting. In addition, by using blocks structure for data storage, XGBoost enables efficient parallel computation. The overall objective function of XGBoost consists of two components: a loss function and a regularization term. Assuming a training set $T=\left\{(x_{1},y_{1}),(x_{2},y_{2}),\cdots,(x_{n},y_{n})\right\}$, a loss function $l(y_{i},\widehat{y_{i}})$, and a regularization term $\Omega(f_{k})$, the objective function can be expressed as . During training, optimization of this objective function yields the optimal weight for each leaf node and the corresponding optimal objective value, The optimal weight is denoted by $w_{j}^{*}=-\frac{G_{j}}{H_{j}\text{+}\lambda}$, the optimal objective value is denoted by $Obj=-\frac{1}{2}\sum_{j=1}^{T} \frac{G_{j}^{2}}{H_{j}\text{+}\lambda}+\gamma T$. Specifically, for a given leaf node $j$, let $G_{j}$ and $H_{j}$ denote the sum of the first-order and second-order derivatives of the loss function for the samples within that node, respectively. A lower objective value $Obj$ indicates a more favorable tree structure. |
| Least Absolute Shrinkage and Selection Operator (LASSO) extends the conventional linear regression model by incorporating an L1 regularization term, which penalizes the absolute values of the regression coefficients. This penalty forces some coefficients to become exactly zero, thereby automatically selecting important features and reducing model complexity. The optimization problem can be formulated as follows: $\arg\min_{\beta} \left(\sum_{i=1}^{N} \left(y_{i}-x_{i}^{T}\beta\right)^{2}+\lambda\left\Vert\beta\right\Vert_{1}\right)$, where $y$ represents the target variable, $\beta$ is the vector of regression coefficients to be estimated, $N$ is the number of samples, and $\lambda$ is the regularization strength parameter. |
| Consensus clustering integrates multiple clustering results obtained via repeated subsampling, thereby reducing the randomness of single clustering and enhancing the overall reliability and robustness. This approach is effective for complex, noisy, or uncertain datasets. The process involves several key steps. First, the original dataset is repeatedly resampled to generate multiple subsets, and each subset is clustered using one or more algorithms (e.g., K-means, hierarchical clustering). Next, a consensus matrix $C$ is constructed, where each entry $C_{ij}$ represents the probability that samples $i$ and $j$ are assigned to the same cluster across all clustering iterations. Finally, techniques such as hierarchical clustering are applied to the consensus matrix to derive the final clustering configuration, selecting the setting that most distinctly partitions the data. |
| Principal Component Analysis (PCA) is a linear transformation technique that projects high-dimensional data onto a low-dimensional orthogonal subspace while preserving maximal variance. Dimensionality reduction is achieved by identifying the directions of greatest variability, known as principal components. In the transformed space, the first PC accounts for the highest variance, with each subsequent PC explaining progressively less. PCA is widely used in genome-wide expression studies to facilitate the analysis of complex, high-dimensional datasets. Let $X_{n\times m}=[x_{1},x_{2},\cdots,x_{m}]$ be a set of $m$ data points, where each $x$ is an n-dimensional column vector. The covariance matrix $C=\frac{1}{m}X^{T}X$ is computed and then decomposed into eigenvalues and eigenvectors. The eigenvectors are sorted in descending order based on their corresponding eigenvalues, and the top $k$ eigenvectors form the transformation matrix $W_{n\times k}$. The new subspace is obtained via the transformation $Y=XW$. |
| T-distributed Stochastic Neighbor Embedding (t-SNE) is a nonlinear dimensionality reduction algorithm particularly suited for projecting high-dimensional data into a lower-dimensional space (typically two or three dimensions) while preserving the essential structure of the original data. It quantifies pairwise similarities in the high-dimensional space using conditional probabilities (denoted as $p_{ij}=\frac{\exp(-\left\Vert x_{i}-x_{j}\right\Vert^{2}/2\sigma^{2})}{\sum_{k\neq i} \exp(-\left\Vert x_{k}-x_{i}\right\Vert^{2}/2\sigma^{2})}$) and similarly defines low-dimensional similarities ($q_{ij}$) using a Student’s t-distribution. The algorithm then minimizes the Kullback-Leibler divergence between these two distributions: $KL(P\vert\vert Q)=\sum_{i} \sum_{j} p_{ij}\log\frac{p_{ij}}{q_{ij}}$. |

## Material 3. Performance evaluation metrics

|  | Predict positive samples | Predict negative samples |
| --- | --- | --- |
| True positive sample | TP | FN |
| True negative sample | FP | TN |

TP: True Positive

FN: False Negative

TN: True Negative

FP: False Positive

| $Accuracy$ | $\frac{TP+TN}{TP+TN+FP+FN}$ |
| --- | --- |
| $Sensitivity(Recall)$ | $\frac{TP}{TP+FN}$ |
| $Precision$ | $\frac{TP}{TP+FP}$ |
| $Specificity$ | $\frac{TN}{TN+FP}$ |
| $F_{1}$ | $\frac{2\times precision\times recall}{precision+recall}$ |

The Receiver Operating Characteristic Curve (ROC curve) is a curve with the false positive rate (FPR) as the horizontal axis and the true positive rate (TPR) as the vertical axis. FPR = 1-specificity = $\frac{FP}{TN+FP}$; TPR = Sensitivity (Recall) = $\frac{TP}{TP+FN}$. The closer the ROC curve is to the top left corner, the better the classification performance of the model is. AUC value is the area under the ROC curve. The larger the AUC value, the better the classification performance of the model.
